# Phage Display Informatics

**DOI:** 10.1155/2013/698395

**Published:** 2013-12-19

**Authors:** Jian Huang, Ratmir Derda, Yanxin Huang

**Affiliations:** ^1^Center of Bioinformatics (COBI), Key Laboratory for NeuroInformation of Ministry of Education, University of Electronic Science and Technology of China, Chengdu 610054, China; ^2^Department of Chemistry and Alberta Glycomics Centre, University of Alberta, Edmonton, AB, Canada T6G 2G2; ^3^National Engineering Laboratory for Druggable Gene and Protein Screening, Northeast Normal University, Changchun 130024, China

Phage display is an efficient laboratory technique that can be used to screen for specific peptides and proteins displayed on the surface of bacteriophage. Since Professor George Smith of the University of Missouri pioneered the powerful and flexible method in 1980s [1], it has been adapted and improved by many scientists from various fields. For example, the sequence displayed on the coat proteins of phage has been extended from random peptides to protein fragments, enzymes, antibodies, and even the whole peptidome of a given species [2]; the way of panning has been expanded from *in vitro* to *in vivo* [3]; the platform for screening has been extended from plates and beads to microfluidic devices [4]. In addition to the development of “hardwares” of phage display, researchers in closely relevant fields have also witnessed the birth and burst of “softwares” for managing enormous amounts of data on phage display and for making biological discoveries or predictions [5, 6]. With the spread of phage display technique and the progress of its “hardwares” and “softwares,” it has made a great impact on modern medicine. For instance, phage display has been widely used for epitope mapping, analysis of protein-protein interactions, prediction of drug target, and identification of enzyme substrates and inhibitors. Some antibodies and peptides derived from phage display technology have been developed into new drugs approved by FDA; others have shown promise for the development of diagnostics, vaccines, and the targeted delivery of therapeutics. In these achievements, informatics means play an increasingly important role.

In this special issue, we take an interest in the investigation of computational and mathematical methods and their applications in all fields using phage display.

For both experimental biologists and computational biologists, mapping conformational B-cell epitopes is a very challenging task. The paper “*Bioinformatics resources and tools for conformational B-cell epitope prediction*” contributed by P. Sun et al. summarized the recent advance of bioinformatics resources and tools for the prediction of conformational B-cell epitopes. According to their review, the prediction methods based on the experimental results of phage display have become one major category of all algorithms. B. He et al. panned the Ph.D.-12 phage display peptide library against metuximab, a new drug for radioimmunotherapy of hepatocellular carcinoma approved by the State Food and Drug Administration of China in 2005, in the paper “*Epitope mapping of metuximab on CD147 using phage display and molecular docking.*” After cleaning their phage display data computationally, they predicted for the first time the complete epitope recognized by metuximab based on the analyses of mimotopes. Very interestingly, the prediction based on phage display largely overlapped with their docking result and the CD147-CD147 interfaces in the CD147 crystal structure. Consequently, they proposed that blocking the formation of CD147 dimer might be an important mechanism of metuximab function. The study by B. He et al. demonstrates that the prediction of conformational B-cell epitopes based on phage display is a cheap and quick strategy with an acceptable accuracy.

Though phage display was born for biomedicine studies, it has already gone beyond this field. For example, it has shown its power in the research for new material, new energy, environmental protection, and agriculture. R. Kushwaha et al. reviewed discoveries via phage display that impacted the use of agricultural products in *“Uses of phage display in agriculture: a review of food-related protein-protein interactions discovered by biopanning over diverse baits.*” Some parts of this review are relevant to medicine and new energy. For instance, the application of phage display in the studies of food allergy and biofuel production was highlighted. Moreover, the utilization of phage display in the defense of plants against herbivores and microbes was discussed. It was expected that phage display and relevant computational methods would become more popular in the agricultural research. Indeed, in another paper “*Uses of phage display in agriculture: sequence analysis and comparative modeling of late embryogenesis abundant client proteins suggest protein-nucleic acid binding functionality.*” by R. Kushwaha et al., sequence analysis and homology modeling were used to study 21 client proteins identified by phage display. The results from this initial computational study would guide their future efforts to uncover the protein protective mechanisms of plant seeds during heat stress.

As we mentioned previously, the blueprint of phage display proposed by Professor George Smith has inspired many scientists to adapt and improve this technique. Different phages and various coat proteins have been tested to construct new phage display systems. As the genomes of hundreds of phages have been sequenced, identification of their virion proteins will be helpful for the development of new phage display systems. P.-M. Feng et al. presented a Naïve Bayes-based method that can predict phage virion proteins using amino acid composition and dipeptide composition in *“Naïve bayes classifier with feature selection to identify phage virion proteins.”* In their jackknife test, the classifier achieved an accuracy of 79.15% to divide phage virion and nonvirion proteins, which were superior to other state-of-the-art methods.

Using next-generation sequencing techniques to enable cost-effective high-throughput analysis is a new trend in phage display technology. However, the trend suffers from errors in deep sequencing data, which may exceed 1%. W. Matochko et al. proposed a linear algebra framework for analyzing errors in a 7-mer peptide library with a medium scale sequenced by Illumina method in *“Error analysis of deep sequencing of phage libraries: peptides censored in sequencing.”* As technical capabilities and depth of sequencing increases, the method would be applicable to larger libraries as well.

In summary, the six papers in this volume involve in various aspects of informatics tools and their applications in several fields using phage display technique. As a snapshot of phage display in the information age, it demonstrates that phage display in the 21st century is being transformed from a purely lab-based science to an information science as well, which can make it even powerful. With the rapid development of “hardwares” and “softwares” of phage display and information technology, we can even expect an in silico phage display system in future.

*Jian Huang* *Yanxin Huang* *Ratmir Derda*
